# Antidiabetic and antihyperlipidemic activity of *Piper longum* root aqueous extract in STZ induced diabetic rats

**DOI:** 10.1186/1472-6882-13-37

**Published:** 2013-02-18

**Authors:** Shaik Abdul Nabi, Ramesh Babu Kasetti, Swapna Sirasanagandla, Thandaiah Krishna Tilak, Malaka Venkateshwarulu Jyothi Kumar, Chippada Appa Rao

**Affiliations:** 1 Department of Biochemistry, Sri Venkateswara University, Tirupati, 517 502 A.P, India; 2 Research Biochemistry, L.V.Prasad Eye Institute, Hyderabad, 500034, India; 3 Department of Biotechnology, Sri Venkateswara University, Tirupati, 517502, India

**Keywords:** Antihypeglycemic, Antihyperlipidemic, Diabetes mellitus, *Piper longum*, Streptozotocin

## Abstract

**Background:**

The available drugs for diabetes, Insulin or Oral hypoglycemic agents have one or more side effects. Search for new antidiabetic drugs with minimal or no side effects from medicinal plants is a challenge according to WHO recommendations. In this aspect, the present study was undertaken to evaluate the antihyperglycemic and antihyperlipidemic effects of *Piper longum* root aqueous extract (PlrAqe) in streptozotocin (STZ) induced diabetic rats.

**Methods:**

Diabetes was induced in male Wister albino rats by intraperitoneal administration of STZ (50 mg/kg.b.w). Fasting blood glucose (FBG) levels were measured by glucose-oxidase & peroxidase reactive strips. Serum biochemical parameters such as glycosylated hemoglobin (HbA1c), total cholesterol (TC), triglycerides (TG), very low density lipoprotein (VLDL), low density lipoprotein (LDL) and high density lipoprotein (HDL) cholesterol were estimated. The activities of liver and kidney functional markers were measured. The statistical analysis of results was carried out using Student t-test and one-way analysis (ANOVA) followed by DMRT.

**Results:**

During the short term study the aqueous extract at a dosage of 200 mg/kg.b.w was found to possess significant antidiabetic activity after 6 h of the treatment. The administration of aqueous extract at the same dose for 30 days in STZ induced diabetic rats resulted in a significant decrease in FBG levels with the corrections of diabetic dyslipidemia compared to untreated diabetic rats. There was a significant decrease in the activities of liver and renal functional markers in diabetic treated rats compared to untreated diabetic rats indicating the protective role of the aqueous extract against liver and kidney damage and its non-toxic property.

**Conclusions:**

From the above results it is concluded that the plant extract is capable of managing hyperglycemia and complications of diabetes in STZ induced diabetic rats. Hence this plant may be considered as one of the potential sources for the isolation of new oral anti hypoglycemic agent(s).

## Background

Diabetes mellitus (DM) is a chronic metabolic disorder characterized by high levels of glucose in the blood due to the impaired secretion of insulin or insulin insensitivity [1]. DM affects approximately 4% of the population worldwide and is expected to increase by 5.4% in 2025 [2]. Hyperglycemia and hyperlipidemia are two important characters of diabetes mellitus. Diabetic patients experience various vascular complications, such as atherosclerosis, diabetic nephropathy and neuropathy [3]. It is now well established that the hyperlipidemia represents a major risk factor for the premature development of atherosclerosis and its cardiovascular complications [4,5]. Currently, the available therapy for diabetes includes insulin and various oral anti-diabetic agents such as sulfonylureas, Thiazolidinediones, α-Glucosidase inhibitors etc. These drugs are used as monotherapy or in combination to achieve better glycemic control. Each of the above oral antidiabetic agents are associated with a number of serious adverse effects [6]. Hence antidiabetic drug discovery has shifted its focus to natural plant sources having minimal side effects. Plants have played a major role in the introduction of new therapeutic agents. A medicinal plant, *Galega officinalis* led to the discovery and synthesis of metformin [7]. Therefore searching herbal product with antidiabetic activity possessing fewer side effects receives considerable publicity and provides an opportunity to cure this disease. Plants play a major role in the discovery of new therapeutic agents and have received much attention as sources of biologically active substances including antioxidants, hypoglycemic and hypolipidemic agents [8]. In pursuit of this goal, several medicinal plants are being investigated for possible hypoglycemic activities based on several approaches including ethanobotanical survey. Of the several indigenous plants used in the local treatment of DM in Rayalaseema region, *Piper Longum* is one of those plants used by tribes to treat diabetes, digestive disorders, obesity etc.

*Piper longum* belongs to family Piperaceae. It grows all over India, in evergreen forests and is cultivated in Assam, Tamil Nadu and Andhra Pradesh. It is a small shrub with a large woody root and numerous creeping, jointed stems, thickened at the nodes. The leaves are alternate, spreading, without stipules and blade varying greatly in size. The fruit, commonly known as pippali and its root, called as pippali mula or modi are used for medicinal purpose. The fruits contain 1% volatile oil, resin, a waxy alkaloid, a terpenoid substance and alkaloids piperine and piperlongumine [9]. There are no reports on the antidiabetic activity of the roots of *Piper longum*. Plants of this genus such as *Piper betle, Piper nigrum* and *Piper sarmentosum* have been reported for their antidiabetic activities [10-12]. Hence this study was taken up to investigate the antihyperglycemic and antihyperlipidemic activities of the roots of *Piper longum* in Streptozotocin (STZ) induced diabetic rats.

## Methods

### Collection of plant material

Dry roots of *Piper longum* (PL), were purchased from the local market and identified by the Botanist, Department of Botany, S.V.University, Tirupati. Voucher specimens (Herbarium Accession Number 713) were deposited in the herbarium, Department of Botany, S.V. University, Tirupati.

### Hexane, ethyl acetate, methanol and aqueous extracts

Hexane, ethyl acetate and methanol extracts were prepared by successive solvent extraction of PL root powder in soxhlet apparatus at 68°C-70°C. The filtrates obtained were distilled and concentrated under reduced pressure at low temperature (40°C to 45°C) in Buchi rotavapor R-200 and finally freeze dried. The yields of the hexane, ethyl acetate and methanol extracts were 38%, 15% and 21% w/w respectively. To prepare aqueous extract the root powder was soaked in distilled water in a glass jar for 48h at room temperature and the solvent was filtered. This was repeated 3–4 times until the filtrate gave no coloration. The filtrate was concentrated to dryness under reduced pressure in Buchi Rotavapor R-200 and finally freeze dried. The yield of the extract was 22% (w/w). All the extracts were stored at 0°C in airtight containers until needed for further studies.

### Induction of diabetes

Diabetes was induced in male Wistar albino rats aged 2–3 months (180–200 g body weight) by intraperitoneal administration of STZ (single dose of 50 mg/kg b.w.) dissolved in freshly prepared 0.01M citrate buffer, pH 4.5 [13]. After 72 h rats with marked hyperglycemia (FBG ≥250 mg/dl) were selected and used for the study. All the animals were allowed free access to tap water and pellet diet and maintained at room temperature in plastic cages.

This study was approved by Institute’s Animal Ethics Committee vide Resolution no: 08/2011-20129(i)/a/CPCSEA/IAEC/SVU/CHA-SAN/dt.25.09.2011.

### Experimental design

#### Evaluation of antihyperglycemic effect of different extracts of *Piper longum* root (Plr) in normal and STZ-induced diabetic rats (Short term study)

The animals were divided into six groups and each group consisted of six rats:

Group 1: Untreated normal rats

Group 2: Untreated diabetic rats

Group 3: Diabetic rats treated with 200 mg Plr.hexane extract /kg b.w.

Group 4: Diabetic rats treated with 200 mg Plr.ethylacetate extract /kg b.w.

Group 5: Diabetic rats treated with 200 mg Plr.methanolic extract/kg b.w.

Group 6: Diabetic rats treated with 200 mg Plr. Aqueous extract /kg b.w.

After an overnight fast the diabetic treated rat groups received the ethyl acetate, methanol, aqueous extracts (dissolved in 1 ml of distilled water) and hexane extract (dissolved in 1 ml of 5% Tween 80) by gastric intubation using a force feeding needle. Untreated normal and diabetic rats were fed distilled water alone. Blood samples were collected from the tail vein at 0, 1, 2, 3, 4, 5 and 6 h after the administration of Plr extracts and blood glucose levels were determined by using glucose oxidase–peroxidise reactive strips.

#### Evaluation of antihyperglycemic activity of PlrAqe in normal and STZ induced diabetic rats - dose dependent study (Short term study)

The animals were divided into 9 groups and each group consisted of six rats:

Group 1: Untreated normal rats

Group 2: Untreated diabetic rats

Group 3: Normal rats treated with 200 mg PlrAqe/kg b.w.

Group 4: Normal rats treated with 300 mg PlrAqe/kg b.w.

Group 5: Normal rats treated with 400 mg PlrAqe/kg b.w.

Group 6: Diabetic rats treated with 200 mg PlrAqe/kg b.w.

Group 7: Diabetic rats treated with 300 mg PlrAqe/kg b.w.

Group 8: Diabetic rats treated with 400 mg PlrAqe/kg b.w.

Group 9: Diabetic rats treated with 0.02 g glibenclamide/kg b.w.

After an overnight fast diabetic treated groups received PlrAqe suspended in distilled water in respective doses, where as untreated normal and diabetic rat groups were fed with distilled water alone with force feeding needle. Blood samples were collected from the tail vein at 0, 1, 2, 3, 4, 5 and 6 h after the administration of PlrAqe and blood glucose levels were determined by using glucose oxidase–peroxidase reactive strips.

Phytochemical analysis was carried out in the PlrAqe by different methods of phytochemical analysis [14].

#### Effect of PlrAqe on oral glucose tolerance (OGT) in normal rats

The rats were divided into three groups, with 6 animals (n = 6) in each group.

Group 1: Normal untreated rats

Group 2: Normal rats treated with 0.02 g glibenclamide/kg b.w

Group 3: Normal rats treated with 200 mg PlrAqe/kg b.w.

After an overnight fast group 2 & Group 3 rats were fed with glibenclamide and PlrAqe respectively. Normal untreated rats (group 1) were fed with distilled water alone. Thereafter, following 30 min of post extract and drug administration all the animals were fed with glucose (2 g/kg.b.wt). Blood samples were collected from tail veins prior to dosing and after 30, 60, 90 and 120 min of glucose administration. FBG levels were analyzed using glucose-oxidase-peroxidase reactive strips (Accu-chek, Roche Diabnostics, GmbH, Germany) [15].

#### Effect of long term treatment with PlrAqe on glycemic control, lipid profile, hepatic and renal function markers in diabetic rats

The rats were divided into 5 groups and each group consisted of 6 rats.

Group 1: Normal untreated rats.

Group 2: Normal rats treated with 200 mg PlrAqe /kg b.w/day.

Group 3: Diabetic untreated rats.

Group 4: Diabetic rats treated with 200 mg PlrAqe /kg b.w/day.

Group 5: Diabetic rats treated with 0.02g of glibenclamide/kg b.w/day.

PlrAqe or glibenclamide was administered to the rats every day morning for 30 days by gastric intubation using oral gavage. Blood samples were collected from tail veins before the start of the treatment and on 10^th^, 20^th^ and 30^th^ days of the treatment and fasting blood glucose levels were estimated. All the five groups of rats were sacrificed on the 30^th^ day after an overnight fast, by anesthetizing with anesthetic ether and further by cervical dislocation and then blood, liver and kidney were collected and immediately stored at -20°C till further analysis. Body weights of all the animals were recorded prior to the treatment and sacrifice.

### Analytical procedures

Estimation of blood glucose was carried out by glucose oxidase–peroxidase method [16]. The estimation of protein was carried out by the Lowry method [17]. HbA1c was estimated by the method of Eross et al. [18]. Estimation of serum cholesterol was carried out by Zlatkis method [19]. Serum triglycerides were estimated by Foster and Dunn method [20] and HDL-cholesterol was estimated by Burstein method [21]. The VLDL cholesterol was calculated using the formula, TG/5 mg/dl. The serum LDL cholesterol was calculated by Friedwald formula [22]. Atherogenic index was calculated by using the formula, TC-HDL-C/HDL-C [23]. Plasma SGOT and SGPT activities were determined by Reitman and Frankel method [24]. Activity of serum alkaline phosphatase (ALP) was determined by p-nitro phenyl phosphate method [25]. Serum creatinine & Serum urea levels were measured by Jaffe’s and diacetyl monoxime methods respectively [26,27].

### Statistical analysis

The results were expressed as mean ± S.D. The statistical analysis of results was carried out using Student t-test and one-way analysis (ANOVA) followed by DMRT.

## Results

### Evaluation of antihyperglycemic effect of different extracts of *Piper longum* root (Plr) in normal and STZ-induced diabetic rats (Short term study)

The effects of hexane, ethyl acetate, methanolic and aqueous extracts of Plr on the fasting blood glucose levels of diabetic rats are given in Table 1. The FBG levels of diabetic untreated rats were significantly higher than those of normal untreated rats (Group 1). When different extracts of Plr were tested for their glucose lowering effects, the methanolic and aqueous extracts at a dosage of 200 mg/kg b.w produced the maximum fall of 30% and 75% respectively, in the FBG levels of diabetic rats after 6 h of treatment. Whereas hexane and ethyl acetate extracts did not show significant antihyperglycemic activity in STZ induced diabetic rats.

**Table 1:** **Effects of Hexane, ethylacetate, methanol and aqueous extracts *of Piper longum *root on FBG levels**

**Group**	**Blood glucose (mg/dl) at different hours after the treatment**						
	**0 hr**	**1 hr**	**2 hr**	**3 hr**	**4 hr**	**5 hr**	**6 hr**
1	87.1 ± 3.0	80.6 ± 6.9	83.6 ± 4.4	84.8 ± 3.7	88.3 ± 5.6	88.1 ± 6.5	88.1 ± 7.9
2	308.1 ± 35.2†	307.8 ± 17.6	352.5 ± 11.3	346 ± 35.87	311.3 ± 29.8	3255.8 ± 22.5	355.8 ± 28.2
3	348 ± 34.3†	336.6 ± 42.3	325.1 ± 47.5	324.8 ± 33.5	342.1 ± 42.9	330.5 ± 32.3	346.8 ± 36.3
4	314 ± 19.8†	309.1 ± 22.8	300.8 ± 40.4	295.1 ± 39	293.1 ± 33.5	307 ± 39.6	292.8 ± 10
5	311.1 ± 15.3†	276.3 ± 22.8	260.5 ± 13*	255.1 ± 19.6*	237.1 ± 14.9**	229.3 ± 29.5** (26%)	209.3 ± 9.3** (30%)
6	348.8 ± 17†	275.3 ± 31*	214.6 ± 24**	153.8 ± 23**	113.6 ± 20** (67%)	94.6 ± 12.2** (72%)	85.5 ± 13.5** (75%)

†P <0.0001 compared with the initial level of blood glucose (0h) of normal rats.

**P <0.0001 compared with the initial level of blood glucose (0h) in the respective group.

* P <0.001 compared with the initial level of blood glucose (0h) in the respective group.

Numbers in parenthesis indicate the percentage of fall in 0h blood glucose.

### Evaluation of antihyperglycemic activity of PlrAqe in normal and STZ induced diabetic rats - dose dependent study (Short term study)

Table 2 shows the dose dependent antihypeglycemic activity of PlrAqe. The FBG levels of diabetic untreated rats were significantly higher than those of normal untreated rats. When different doses of PlrAqe were tested for their glucose lowering effects, the aqueous extract at a dosage of 200 mg/kg b.w produced the maximum (75%) fall in the FBG levels of diabetic rats after 6 h of treatment, while the doses 300 and 400 mg/kg bw produced 51% and 25% fall in the FBG levels respectively after 6 h of treatment. None of the doses of the PlrAqe caused any hypoglycemic activity in normal treated rats. Treatment with glibenclamide at a dosage of 0.02 g/kg b.w of diabetic rats resulted in 35% fall in FBG after 5 h of treatment.

**Table 2:** Effect of different doses of PlrAqe on FBG levels of normal and diabetic rats

**Group**	**Blood glucose (mg/dl) at different hours after the treatment**						
	**0 hr**	**1 hr**	**2 hr**	**3 hr**	**4 hr**	**5 hr**	**6 hr**
1	76.5 ± 4.9	76.5 ± 6.1	74.5 ± 4.5	73 ± 4.7	78.3 ± 6.8	73.8 ± 5.9	76.8 ± 5.7
2	347 ± 39†	368 ± 45.4	374.5 ± 52	403 ± 68.9	405.5 ± 36	337 ± 38	423 ± 57
3	83.5 ± 10	82.3 ± 10.7	79.3 ± 7.7	80 ± 2.9	81.1 ± 6.1	77.5 ± 12	77 ± 10.2
4	80 ± 7.9	80.5 ± 11.7	75.8 ± 7.3	78.5 ± 9.7	81 ± 10.7	79 ± 13.8	82.5 ± 12
5	77 ± 12	77.1 ± 7.5	74.3 ± 6.5	74 ± 1	77.5 ± 7.1	71 ± 5.8	73 ± 3.1
6	348.8 ± 17†	275.3 ± 31*	214.6 ± 24**	153.8 ± 23**	113.6 ± 20**	94.6 ± 12.2** (72%)	85.5 ± 13.5** (75%)
7	358.5 ± 38†	271.6 ± 16*	210.3 ± 24**	202.5 ± 20**	184 ± 22**	177 ± 14** (50%)	172 ± 16** (51%)
8	397 ± 43†	374.8 ± 30	357.5 ± 31	339.5 ± 34	320.3 ± 35	299.6 ± 34* (24%)	295.5 ± 34* (25%)
9	322 ± 34†	293.8 ± 10.7	265.3 ± 24	243.5 ± 24*	216 ± 21.4**	208 ± 14.9** (35%)	233 ± 23.5** (27%)

† P <0.0001 compared with the initial level of blood glucose (0h) of normal rats.

** P <0.0001 compared with the initial level of blood glucose (0h) in the respective group.

* P <0.001 compared with the initial level of blood glucose (0h) in the respective group.

Numbers in parenthesis indicate the percentage of fall in 0h blood glucose.

Phytochemical analysis revealed the presence of glycosides, alkaloids and carbohydrates in PlrAqe.

### Effect of PlrAqe on oral glucose tolerance (OGT) in normal rats

**Figure 1:**
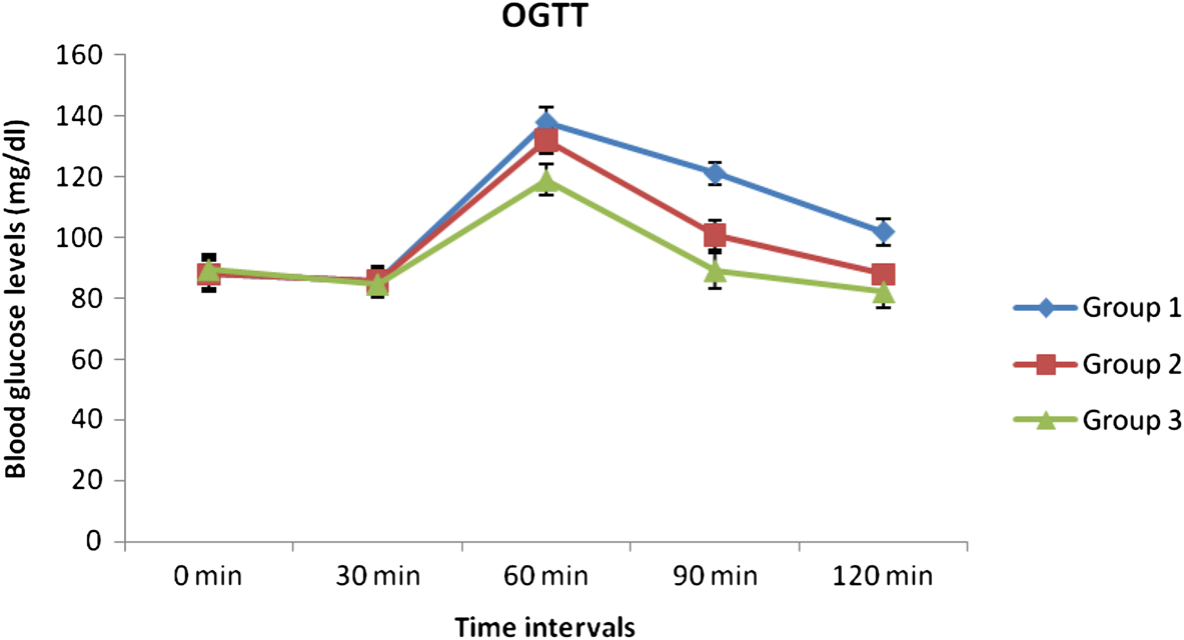
Effect of PlrAqe on oral glucose tolerance in normal rats.

In this study the FBG levels of all groups of animals were estimated from 0 min to 120 min. In all groups the FBG levels were raised at 60 min (due to glucose load) but after that there was a significant decrease in the FBG levels of group II and III when compared to group I. The results are depicted in Figure 1.

### Effect of long term (30 days) treatment with PlrAqe on hyperglycemia, HbA_1C_ and body weights of diabetic and normal rats

PlrAqe exhibited significant antihyperglycemic activity after 30 days of treatment in diabetic rats. It produced 66.7% (P < 0.001) fall in FBG levels compared to the initial FBG levels prior to the treatment. No significant effect was observed in normoglycemic rats. Glibenclamide produced 33% decrease in FBG levels in diabetic rats. At the end of 30 days treatment, the HbA_1C_ levels of the diabetic untreated group were significantly higher than those in normal control group (11.18 ± 2.0% and 5.73 ± 0.47% respectively). Treatment with the PlrAqe in diabetic rats reduced the HbA_1C_ to a significant level (7.1 ± 0.46%), indicating a significant improvement in glycemic control in diabetic rats upon treatment. The body weights of normal, normal treated, diabetic treated and glibenclamide treated group, increased significantly by +25.8 g, +31.6 g, +24.1 g and +21.2 g respectively, whereas the body weights of diabetic control group decreased by −33.3 g (Table 3).

**Table 3:** Effect of long term treatment with the PlrAqe on hyperglycemia, Hb, HbA1c and body weights

**Group**	**Blood glucose (mg/dl) at different days during the experimental period**						
	**1**^**st**^**Day**	**10**^**th**^**Day**	**20**^**th**^**Day**	**30**^**th**^**Day**	**Hb (g/dl)**	**HbA**_**1c**_**(%)**	**Change in body weights (g)**
1	87.1 ± 7.3^a^	93.5 ± 7.5^a^	89.3 ± 8.5^a^	85.1 ± 6.7^a^	11.1 ± 1.36^b^	5.73 ± 0.47^a^	+25.8 ± 4.3^a,b^
2	77 ± 5.3^a^	79.1 ± 7.2^a^	79 ± 9.3^a^	83.1 ± 6.2^a^	11.16 ± 1.04^b^	5.48 ± 1.2^a^	+31.6 ± 3.8^b,c^
3	357 ± 14.98^b^	424 ± 21^d^	433 ± 29^c^	449 ± 24^c^	7.166 ± 0.91^a^	11.18 ± 2.0^b^	-33.3 ± 7.5^c^
4	319 ± 30^b^	188 ± 17^b^	106 ± 17.3^a^	106 ± 21.7^a^	10.71 ± 0.83^b^	7.1 ± 0.46^a^	+24.1 ± 2.4^a^
5	312.5 ± 28.1^b^	277 ± 33^c^	249.5 ± 17^b^	208 ± 20.9^b^	10.25 ± 0.74^b^	7.2 ± 0.64^a^	+21.2 ± 2.6^a^
F Value	270.185	309.482	419.650	448.880	16.656	23.733	6.364
Significance	0.000	0.000	0.000	0.000	0.000	0.000	0.002

Values are given as mean ± S.D from six rats in each group.

Values not sharing a common superscript letter differ significantly at p < 0.01 (DMRT).

### Effect of long term treatment with PlrAqe on hyperlipidemia, hepatic and renal function markers

**Figure 2:**
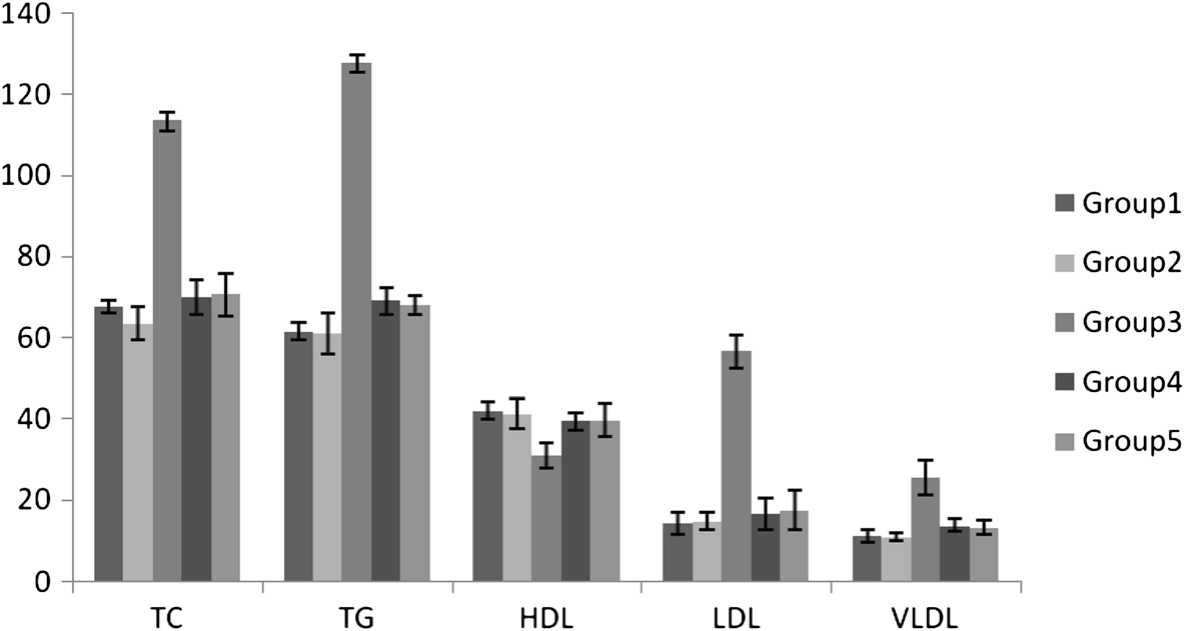
Effect of PlrAqe on serum total cholesterol, triglycerides, HDL, LDL and VLDL cholesterol in normal and experimental groups of rats after 30 days treatment.

Figure 2 shows the serum levels of TC, TG, LDL, VLDL and HDL cholesterol in normal and experimental animals in each group. The diabetic untreated group had significant elevation of TC, TG, LDL, VLDL and reduction in HDL-C levels as compared to the normal control rats. A significant reduction in TC, TG, LDL, VLDL and increase in HDL-C levels were observed in diabetic rats treated with either PlrAqe or glibenclamide. The atherogenic index in diabetic untreated rats was much higher than that of normal rats. Treatment of diabetic rats with PlrAqe provided 70.8% protection against atherogenicity, by decreasing the atherogenic index significantly (Table 4).

**Table 4:** Effect of PlrAqe on atherogenic index and percent of protection against atherogenicity caused by experimental diabetes

**Group**	**Atherogenic index (AI)**	**Percent of protection from atherogenicity**
1	0.605	
2	0.537	
3	2.649	
4	0.772	70.8%
5	0.782	

**Figure 3:**
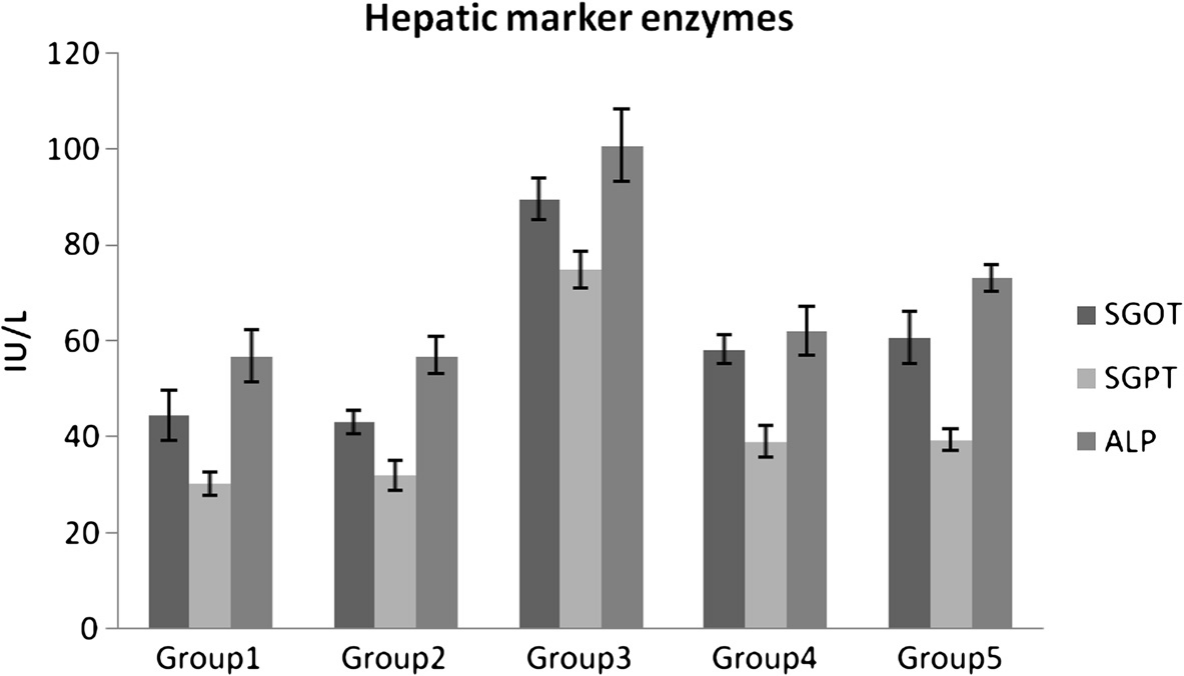
Effect of the PlrAqe on the liver marker enzymes SGOT, SGPT and ALP in normal and experimental diabetic animals.

Figure 3 and Table 5 show the levels of hepatic and renal functional markers in all experimental rat groups respectively. Diabetic rats showed elevated activities of hepatic (SGOT, SGPT and ALP) and renal (serum urea & creatinine) functional markers. The above enzyme activities were maintained near to normal levels in diabetic treated group. Similar effects were observed with glibenclamide. There were no significant changes in the levels of hepatic and renal function markers in the normal treated rats.

**Table 5:** Effect of PlrAqe on the plasma urea and creatinine levels

**Groups**	**UREA (mg/dl)**	**CREATININE (mg/dl)**
1	41.7 ± 3.2^a^	0.424 ± 0.028^a^
2	40.9 ± 6.02^a^	0.40 ± 0.02^a^
3	63.6 ± 4.2^b^	0.819 ± 0.03^c^
4	44.1 ± 3^a^	0.597 ± 0.016^a^
5	47.4 ± 6.6^a^	0.482 ± 0.11^ab^
F - Value	21.909	52.533
Significance	0.000	0.000

Values are given as mean ± S.D from six rats in each group.

Values not sharing a common superscript letter differ significantly at p < 0.01 (DMRT).

## Discussion

Sheshachala forest (Rayalaseema region, Andhra Pradesh, India), which lie geographically in the South Eastern Ghats are known for the rich heritage of flora where the tribes use *Piper longum* roots to treat the diabetes mellitus [28]. Hence our study was aimed to find out the scientific evidence for the safe use of the roots of *Piper longum* to treat DM. Earlier, Shanmugam Manoharan *et al.* demonstrated that the ethanolic extract of dried fruits of *Piper longum* has potent antihyperglycemic and antilipidperoxidative activity in alloxan induced diabetic rats [29]. In the present study, we have selected the roots of *Piper longum* for evaluating antihyperglycemic and antihyperlipidemic activity since the phytochemical constituents of fruits and roots are different. In our study STZ was used to induce diabetes mellitus in rats. At low dose, STZ (50 mg/kg b.w) partially destructs the beta cells resulting in insufficient insulin secretion causing type 2 diabetes [30]. It is widely accepted animal model and reported to resemble human hyperglycemic non ketotic diabetes mellitus [31], is often associated with kidney hypertrophy which may contribute to end stage renal damage, hepatotoxicity, oxidative stress and hypercholesterolemia [32,33].

During the short term study the aqueous and methanolic extracts produced significant antihyperglycemic activity at a dosage of 200 mg/kg b.w in diabetic treated rats. Aqueous extract is more potent than methonolic extract. The hexane and ethylacetate extracts did not show significant antihyperglycemic activity, may be due to the lack of phytochemical constituents like alkaloids and glycosides which are present in methanolic and aqueous extracts.

During the dose dependent study, the aqueous extract at a dosage of 200 mg/kg b.w has produced maximum antihyperglycemic activity in diabetic rats. The higher doses of PlrAqe (300 & 400 mg/kg b.w) could not produce the predictable higher antihyperglycemic effect due to the presence of some other substances in the aqueous extract, which interfere with the antihyperglycemic effect [34]. So, the long term study was carried out with the dose of 200 mg PlrAqe/kg b.w.

The oral glucose tolerance test also confirmed blood glucose lowering activity of PlrAqe. The onset of antihyperglycemic action was observed from 60 min of the treatment and a steady state increase in the action continued up to 120 min. The PlrAqe may be involved in enhancement of glucose utilization, so blood glucose levels were significantly decreased in glucose loaded rats.

The loss in body weights observed in STZ induced diabetic rat group (after a period of 30 days) may be due to muscle wasting and loss of tissue proteins upon induction of diabetes with STZ [35,36]. The gain in body weight was observed both in normal treated and diabetic treated groups. PlAqe treatment in diabetic treated group for 30 days resulted in a significant (66%) reduction in their FBG levels and these effects were higher than those of the standard oral hypoglycemic agent glibenclamide. Earlier Santhakumari *et.al*[37] and Kaleem *et.al*[11] reported 26% and 52% reduction in the FBG levels of diabetic rats treated with aqueous extracts of *Piper betle* leaves and *Piper nigrum* seeds respectively for 30 days.

HbA1c is used as a marker for estimating the degree of protein glycation in diabetes mellitus. HbA1c was found to increase in patients with diabetes mellitus and the amount of increase is directly proportional to the fasting blood glucose level [38]. In diabetic condition, the excess glucose present in the blood reacts with haemoglobin to form HbA1c [39]. Hence HbA1c levels were elevated and total haemoglobin levels were depleted in untreated diabetic rats. HbA1c levels were well regulated near to normal levels in PlrAqe treated diabetic group, this could be due to an improvement in insulin secretion upon PlrAqe treatment. *Piper betle* which belongs to same genus has been reported to have the ability to reduce HbA1_C_ levels in diabetic rats [37].

Diabetes mellitus is usually associated with prominent levels of serum lipids and such an increase causes the risk factor for coronary heart diseases [40]. A variety of alterations in metabolic and regulatory mechanisms, due to insulin deficiency or due to insulin resistance are responsible for the observed accumulation of lipids [41]. STZ-induced diabetes also developed hyperlipidemia which is in agreement with our previous observations [34,42]. In the present study, the PlrAqe significantly reduced the TC, TG, LDL-C and VLDL-C levels with an increase of HDL-C in treated diabetic rats compared to untreated diabetic rats (Figure 2). This may be due to the insulinotropic effect or insulin secretagogue activity of this extract. PlrAqe treated diabetic rats showed decrease in atherogenic index and increase in percentage of protection against atherogenicity. Decrease in atherogenic index is due to increase in HDL-C levels after the treatment. HDL-C is known to play an important role in the transport of cholesterol from peripheral cells to the liver by a pathway termed reverse cholesterol transport, and is considered to be a cardio protective lipid. The existence of negative correlation between HDL-C and atherosclerosis resulted in improvement in the percentage of protection against atherogenicity in STZ induced diabetic treated rats [43].

Under hyperglycemic condition disturbances in carbohydrate, lipid and protein metabolisms together with oxidative stress are likely to affect hepatic and renal functions. Hence our study was also focused to know the protective activity of PlrAqe against hepatic and renal damage caused by diabetes. In the present study serum enzymes such as SGOT, SGPT and ALP were used in the evaluation of hepatic damage (Figure 3). In diabetic rats an increase in these enzyme activities reflects active liver damage. Increased levels of SGOT and SGPT under insulin deficiency [44] have been related with increased gluconeogenesis and ketogenesis during diabetes. Moreover, increased levels of these enzymes together with ALP and Acid phosphatase (ACP) are reported to be associated with liver dysfunction and leakage into blood stream in diabetes [45]. Oral administration of PlrAqe in diabetic rats resulted in reduction in the activities of these enzymes in serum compared to the diabetic untreated group.

In our study elevated levels of serum urea and creatinine were observed in diabetic untreated rats, which are considered as significant markers of renal dysfunction [46]. Negative nitrogen balance with enhanced tissue proteolysis and decreased protein synthesis can contribute to increased serum urea and creatinine levels, indicating impaired renal functions in diabetic animals [47]. After the treatment with PlrAqe a significant reduction in the levels of urea and creatinine were observed in the diabetic treated rats. It indicates that PlrAqe is preventing the renal damage in diabetic rats.

## Conclusion

All these beneficial effects of PlrAqe are especially hopeful in preventing hyperglycemia, cardiovascular, hepatic and renal diseases. In conclusion, this study has undoubtedly provided scientific confirmation and evidence for the safe use of the roots of *Piper longum* by traditional healers in the treatment of diabetes. However the nature of the active principle(s) responsible for all these positive effects requires further investigation.

## Abbreviations

PlrAqe: *Piper longum* root aqueous extract; STZ: Streptozotocin; FBG: Fasting blood glucose; HbA1c: Glycosylated hemoglobin; TC: Total cholesterol; TG: Triglycerides; VLDL: Very low density lipoprotein; LDL: Low density lipoproteins; HDL: High density lipoproteins; Plr: *Piper longum* root; OGTT: Oral glucose tolerance test.

## Competing interest

All authors are in agreement with the content of the manuscript and authors do not have any conflict of interest.

## Authors’ contributions

SAN has made significant contribution throughout the study starting from collection of *Piper longum* roots to the completion of the study. RBK participated in planning the experiments, evaluation of anti hyperglycaemic activity and statistical analysis. SS, TKT and MVJK maintained the animals and helped in estimation of lipid profiles, liver & renal functional markers. CA designed the whole study, analyzed & interpreted the results and corrected the manuscript. All authors read and approved the final manuscript.

## Pre-publication history

The pre-publication history for this paper can be accessed here:

http://www.biomedcentral.com/1472-6882/13/37/prepub
